# The herbicides glyphosate and glufosinate and the cyanotoxin β-N-methylamino-l-alanine induce long-term motor disorders following postnatal exposure: the importance of prior asymptomatic maternal inflammatory sensitization

**DOI:** 10.3389/fnins.2023.1172693

**Published:** 2023-06-09

**Authors:** Asma Oummadi, Arnaud Menuet, Sarah Méresse, Anthony Laugeray, Gilles Guillemin, Stéphane Mortaud

**Affiliations:** ^1^Experimental and Molecular Immunology and Neurogenetics, UMR7355 CNRS, Orléans, France; ^2^Faculty of Medicine and Human Health Sciences, Center for MND Research, Macquarie University, Sydney, NSW, Australia; ^3^UFR Sciences et Techniques, University of Orléans, Orléans, France; ^4^Faculty of Biology and Medicine, Department of Fundamental Neurosciences, Lausanne, Switzerland

**Keywords:** exposome, multiple-hit, asymptomatic inflammation, perinatal exposure, environmental pollutants, neurodevelopment

## Abstract

**Background:**

Prenatal maternal immune activation (MIA) and/or perinatal exposure to various xenobiotics have been identified as risk factors for neurological disorders, including neurodegenerative diseases. Epidemiological data suggest an association between early multi-exposures to various insults and neuropathologies. The “multiple-hit hypothesis” assumes that prenatal inflammation makes the brain more susceptible to subsequent exposure to several kinds of neurotoxins. To explore this hypothesis and its pathological consequences, a behavioral longitudinal procedure was performed after prenatal sensitization and postnatal exposure to low doses of pollutants.

**Methods:**

Maternal exposure to an acute immune challenge (first hit) was induced by an asymptomatic lipopolysaccharide (LPS) dose (0.008 mg/kg) in mice. This sensitization was followed by exposing the offspring to environmental chemicals (second hit) postnatally, by the oral route. The chemicals used were low doses of the cyanotoxin β-N-methylamino-l-alanine (BMAA; 50 mg/kg), the herbicide glufosinate ammonium (GLA; 0.2 mg/kg) or the pesticide glyphosate (GLY; 5 mg/kg). After assessing maternal parameters, a longitudinal behavioral assessment was carried out on the offspring in order to evaluate motor and emotional abilities in adolescence and adulthood.

**Results:**

We showed that the low LPS immune challenge was an asymptomatic MIA. Even though a significant increase in systemic pro-inflammatory cytokines was detected in the dams, no maternal behavioral defects were observed. In addition, as shown by rotarod assays and open field tests, this prenatal LPS administration alone did not show any behavioral disruption in offspring. Interestingly, our data showed that offspring subjected to both MIA and post-natal BMAA or GLA exposure displayed motor and anxiety behavioral impairments during adolescence and adulthood. However, this synergistic effect was not observed in the GLY-exposed offspring.

**Conclusion:**

These data demonstrated that prenatal and asymptomatic immune sensitization represents a priming effect to subsequent exposure to low doses of pollutants. These double hits act in synergy to induce motor neuron disease-related phenotypes in offspring. Thus, our data strongly emphasize that multiple exposures for developmental neurotoxicity regulatory assessment must be considered. This work paves the way for future studies aiming at deciphering cellular pathways involved in these sensitization processes.

## 1. Introduction

Early life events play an important role in the health and neurodevelopment of an individual. Throughout life, from the embryonic period until aging, an individual is exposed to a multitude of environmental agents, through various sources such as our diet, pollutants, or microorganisms. During *in-utero* stages, the maternal environment affects the growing fetus. It is well documented that maternal immune activation (MIA), induced by infectious and non-infectious insults, is a serious risk factor for subsequent neuropathologies in offspring, whereby increasing the level of maternal pro-inflammatory cytokines and interfering with fetal brain development (Meyer, 2014; Estes and McAllister, 2016). MIA has been principally described as an inductor of neuropsychiatric disorders such as neurodevelopmental disorders (Choudhury and Lennox, 2021; Han et al., 2021), but could also be a major factor contributing to neurodegenerative diseases (Tartaglione et al., 2015; Nisa et al., 2021). However, the majority of maternal infections do not induce permanent disorders in offspring (Estes and McAllister, 2016), suggesting other additional disruptive factors.

Among them, exposure to agricultural pesticides during pregnancy and/or lactation could trigger the onset of neuronal adult disorders (Rauh et al., 2012; Ayeni et al., 2022). In fact, experimental studies highlighted the association between several highly used and frequently studied pesticides (e.g., glyphosate) during the perinatal period and disruptive long-term effects in offspring. Thus, Gallegos et al., have shown a delay in sensorimotor reflexes acquisition and altered locomotor activity and anxiety behavior in rat offspring following perinatal exposure to glyphosate-based herbicide (GBH) as well as an impairment in recognition memory (Gallegos et al., 2016, 2018). Further investigations revealed that these behavioral defects were associated with cholinergic/dopaminergic system dysfunction and oxidative stress impairments in the offspring (Ait-Bali et al., 2020; de Oliveira et al., 2022). Additionally, other work demonstrated that glyphosate exposure during the prenatal period leads to motor and cognitive defects in pups, suggested to be linked to the downregulation of the Wnt/Ca^+2^ pathway (Coullery et al., 2020). In line with these findings, other types of herbicides have been also incriminated. Thus, we previously demonstrated that perinatal exposure to a low dose of another well-known herbicide, glufosinate ammonium (GLA), led to autistic-like behavioral phenotype and neuroblastic migration disturbances (Laugeray et al., 2014; Herzine et al., 2016). More recently, another analysis revealed that perinatal GLA exposure led to impaired motor coordination functioning in offspring, as well as affecting cortical development, in particular interneuron migration (Kim et al., 2020). Dong and collaborators have also demonstrated reduced locomotor activity and impaired memory in a mouse model exposed to GLA prenatally (Dong et al., 2020).

In addition, among environmental toxicants, an increasing number of studies put forward the involvement of non-anthropogenic compounds as a potential etiological factor of many neurological disorders. β-N-Methylamino-l-alanine (BMAA), a cyanotoxin synthetized by cyanobacteria, is increasingly considered as a potential environmental risk factor for sporadic neurodegenerative outcomes, such as amyotrophic lateral sclerosis (ALS), Alzheimer’s disease (AD) or Parkinsonism-dementia complex (PDC) (Pablo et al., 2009; Caller et al., 2018; Cox et al., 2018). Interestingly, a BMAA post-natal exposure performed in rats induced neurodevelopmental neurotoxicity observed through specific learning impairments (Karlsson et al., 2009b). More recently, we demonstrated, with an extended exposure window including the prenatal period, short and long-lasting emotional disturbances in offspring (Laugeray et al., 2018).

Despite the great diversity of experimental procedures using a wide range of doses associated with different treatment durations and exposure periods, it appears that the perinatal period is a particularly sensitive window for the emergence of nervous system disturbances. The pre-and post-natal periods are highly complex and essential time spans for brain development, during which extremely controlled phenomena occur, such as neurogenesis or differentiation and maturation of neuronal precursors (Purves et al., 2012). Modifications to these tightly regulated processes may be responsible for permanent impairments, thereby leading to a wide range of enduring adverse impacts later in life. This is the core principle of the Barker hypothesis better known as the Developmental Origins of Health and Disease (DOHaD) (Sinclair and Singh, 2007). Several hypotheses have been made, including that environmental agents epigenetically deregulate developmental gene expression, disturbing their functions in a long-term manner (Lahiri and Maloney, 2012). Similarly, recent data demonstrated that early exposure to pollutants might disrupt microbiome composition that is able to disturb neurodevelopmental processes (Stiemsma and Michels, 2018).

Thus, these already published studies underline that each individual could be exposed to a variety of factors that define their exposome. This concept, introduced by Christopher Wild in 2005, was born of the need to better understand the health impact of all environmental exposures (e.g., pollutants) an individual could be exposed to, from conception to death, complementing genome-induced effects (Wild, 2005). While multiple exposures may increase the risk of developing major mental illnesses and neurodegenerative disorders, it is possible that there is an epidemiological association between exposure to various insults during pregnancy and neurological disorders. Thus, based on the principle of the “multiple-hit hypothesis,” prenatal inflammation makes the brain more susceptible to subsequent exposures to several kinds of neurotoxins. MIA would be a “disease primer” in the brain’s responsiveness to cumulative lifetime exposure to environmental insults (Kristin et al., 2019).

Taken together, our hypothesis is that an acute and asymptomatic low-dose MIA may act synergistically with low-dose pesticides/cyanotoxin exposure to trigger behavioral disruptions in offspring, leading to neurological disease development later in life. To explore the “multiple-hit hypothesis,” we investigated the behavioral effects of early exposure to an acute immune challenge (first hit) induced by an asymptomatic lipopolysaccharide (LPS) dose (Arsenault et al., 2014), alongside an evaluation of maternal behavior and the release of pro-inflammatory cytokines. This sensitization was associated with the offspring’s post-natal exposure, by the oral route, to environmental chemicals (second hit), i.e., low doses of BMAA, GLA, or glyphosate (GLY). A longitudinal behavioral assessment was conducted on the offspring in order to evaluate motor and emotional abilities in adolescence and adulthood.

## 2. Materials and methods

### 2.1. Animals, treatment, and general procedure

**Figure 1:**
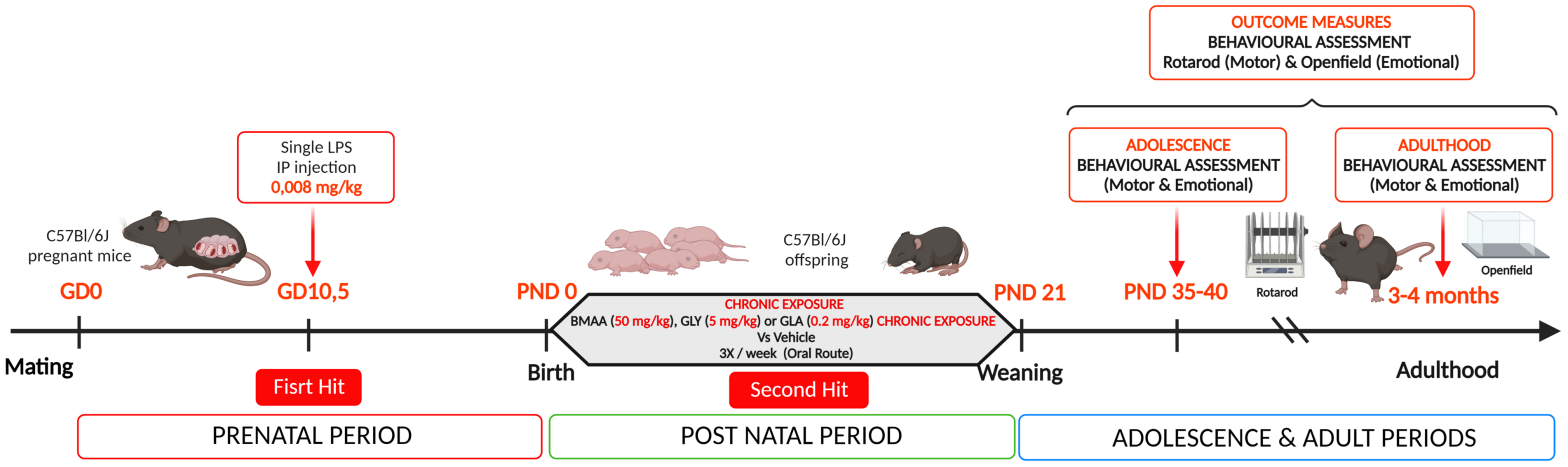
Experimental design used in the study. A prenatal immune challenge has been made at gestational day 10.5 in pregnant C57Bl/6 J mice with a single intraperitoneal (IP) injection of lipopolysaccharide at a dose of 0.008 mg/kg – First Hit (*n* = 19 saline (Sal) and *n* = 18 LPS). This maternal sensitization was followed by a postnatal chronic exposure, via the oral route, to environmental compounds: BMAA (50 mg/kg), GLY (5 mg/kg), or GLA (0.2 mg/kg) – Second Hit (*n* = 10 Sal/CTL, *n* = 9 Sal/BMAA, *n* = 8 Sal/GLA, *n* = 9 Sal/GLY, *n* = 7 LPS/CTL, *n* = 9 LPS/BMAA, *n* = 9 LPS/GLA and *n* = 7 LPS/GLY). Outcome measures consisted of a longitudinal behavioral assessment, during adolescence and adulthood. Motor learning and motor coordination behavior were assessed by the rotarod task and locomotor and emotional behavior with the open field test.

For the experimental procedure, 60 five-week-old female C57Bl/6J mice from Janvier Labs (Le Genest-St-Isle, France) were initially used. The animals were housed and maintained on a 12 h light/dark cycle with *ad libitum* access to food and drink, at 21 ± 1°C, and humidity 50–70% in an EOPS resource facility (UPS44, CNRS, Orléans, France). After three weeks of acclimation, the C57Bl/6 J mice were bred (two females and one male), in standard Polycarbonate-PC cages (365 × 207 × 140 mm). Every morning (between 7:30 and 8:00 AM), females were checked for the presence of a vaginal plug to determine if they had been successfully mated during the night, thereby corresponding to gestational day (GD) 0. Pregnant mice were then isolated and randomly divided into two groups, which were administered either LPS 0.008 mg/kg body weight (L2880-10MG - Lipopolysaccharides from *Escherichia coli* O55:B5, Merck Sigma-Aldrich) or saline solution (NaCl 0.9%) *via* intraperitoneal (IP) injection at GD 10.5 (Hit 1) (Figure 1). From GD0, dams were weighed every two days until delivery. This experiment involved 37 pregnant females: 19 saline (Sal) and 18 LPS. In order to verify whether LPS sensitization induces physiological and behavioral changes in dams, body weight was measured every 2 days during the prenatal period. Maternal behavior was assessed through the dams’ ability to retrieve their pup at post natal day (PND) 3.

The number of male and female offspring was determined 1 day after birth and it was decided to choose two male pups at random per litter for subsequent adolescent and adult behavioral assessments. The two males were identified *via* toe-clipping methods at PND4. The offspring were exposed *per os*, three times a week, from PND4 to weaning with either BMAA 50 mg/kg bw (B107-50MG, 16,012–55-8, ≥97% (NMR), Merck Sigma-Aldrich), GLA 0.2 mg/kg bw (45520-100MG, 77,182–82-2, PESTANAL^®^) or GLY 5 mg/kg bw (45521-250MG, 1,071-83-6, PESTANAL^®^) (Hit 2) (Figure 1). All the compounds were prepared in a saline solution of 0.9% NaCl. Control animals received a comparable dose of the vehicle solution. For practical reasons, we used a 20 μL pipette (P20 with the appropriate tips) to administer the drinkable oral solution directly into the pup’s mouth. As offspring exposure was carried out orally. The litters in the saline and LPS groups were separated depending on the type of post-natal exposure (BMAA GLA or GLY) to avoid contaminating other pups with a different treatment *via* urine production, licking, or suckling. The saline group included: 5 control litters (CTL) (Sal/CTL), 5 BMAA litters (Sal/BMAA), 4 GLA litters (Sal/GLA), and 5 GLY litters (Sal/GLY). The LPS group included: 4 control litters (LPS/CTL), 5 BMAA litters (LPS/BMAA), 5 GLA litters (LPS/GLA), and 4 GLY litters (LPS/GLY).

At PND21, the offspring were weaned. Labeled males were regrouped per type of pre-and post-natal treatment. Shortly after the end of the treatment, approximately PND 40, and later during adulthood (3–4 months), a longitudinal behavioral assessment was performed. Two behavioral spheres were assessed: motor learning and motor coordination behavior using the rotarod task, and locomotor and emotional behavior with the open field test. This experiment involved: *n* = 10 Sal/CTL, *n* = 9 Sal/BMAA, *n* = 8 Sal/GLA, *n* = 9 Sal/GLY, *n* = 7 LPS/CTL, *n* = 9 LPS/BMAA, *n* = 9 LPS/GLA, and *n* = 7 LPS/GLY.

For cytokine level measurement, 30 female C57Bl/6 J wild-type mice were obtained from Janvier Labs (Le Genest St Isle, France) and were bred as described above. Mating and procedure to determine if the mice were pregnant were also the same as previously described. Once a vaginal plug had been identified, pregnant mice were weighed every two days until GD 10.5. In total, 23 pregnant mice were obtained and were randomly and equally (as far as possible) assigned to three experimental groups which were administered either LPS 0.008 mg/kg body weight (bw) (L2880-10MG - Lipopolysaccharides from *Escherichia coli* O55:B5, Merck Sigma-Aldrich), LPS 0.1 mg/kg bw or saline solution (NaCl 0, 9%) *via* intraperitoneal (IP) injection at GD 10.5. The dams were euthanized using an anesthetic overdose of isoflurane (beginning with an isoflurane flow rate of 7% and continuing isoflurane exposure until two minutes after breathing stopped), and mouse peritoneal cell suspensions were collected six hours after the LPS or saline IP injection. This experiment involved in a total of 23 pregnant females (*n* = 23) spread into three distinct groups: Saline (Sal; *n* = 7), LPS Low (0.008 mg/kg bw; *n* = 8), and LPS High (0.1 mg/kg bw; *n* = 8).

All animal care and experimentation were in accordance with the European Communities Council directive (2010/63/EU) and approved by the local Ethics committee (Approval C45-234-6).

### 2.2. Maternal behavior assessment: pup retrieving test

This task was performed to verify whether maternal behavior is modified by a mild inflammatory episode during pregnancy. The retrieval ability was measured by the mother’s latency to retrieve the first pup and the total time required to retrieve three pups at PND3. The mother was removed from her home cage for 1 min. Three pups were removed from the nest and then placed in each corner of the cage. The mother was repositioned in her nest, and both the latency to retrieve the first pup and the time to retrieve all three pups were recorded. Retrieval was counted only when the pup was brought fully into the nest, and a score of 180 s was assigned if the dam failed to retrieve her pups within 3 min.

### 2.3. Adolescent and adult behavior: rotarod (accelerated mode)

**Figure 2:**
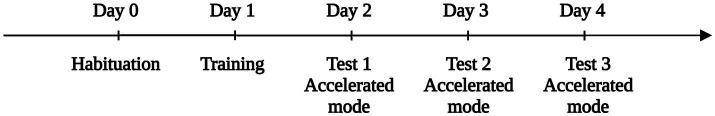
Rotarod testing procedure.

The rotarod test is used to assess motor coordination and balance in mice. This test was carried out during adolescence (approximately PND-40) and during adulthood (approximately 3–4 months). The procedure consisted of 2 distinct phases over 4 consecutive days (Figure 2). One day prior to the test, home cages were placed in the experimental room with the rotarod device turned on in order to habituate mice to the experimental environment and reduce the effects of stress on their behavior during testing (Day 0).

The first day (Day 1) corresponded to the training phase. Animals were placed on the rotating rod fixed at a speed of 4 rpm until they walked forwards to keep balance. A 60-s cutoff was defined. Once 60 s was reached, mice were replaced in their home cage and the apparatus was deep-cleaned with 70% ethanol and water. The procedure was repeated for 3 trials separated by 5 min intersession intervals. Mice had to be capable of staying on the rod for 60 s before proceeding to testing.

The three following consecutive days (Days 2, 3, and 4) corresponded to the testing phase. The animals were placed on the rod then the apparatus was set to accelerate from 4 to 40 rpm in 5 min. The trial began when the device began accelerating and stopped when the subject fell off the rod. Latency to fall and the rpm associated were the parameters collected for analysis. The procedure was repeated for 4 trials separated by a 3 min intersession interval. After each session, the apparatus was deep-cleaned with 70% ethanol and water.

### 2.4. Adolescent and adult behavior: open field

The open field task was used to assess the ability of mice to deal with unescapable stressful situations. This test was carried out during adolescence (approximately PND-40) and during adulthood (approximately 3–4 months). The setup consisted of a 60 cm (length [L]) x 60 cm (width [W]) x 40 cm (height [H]) nontransparent gray Plexiglas arena. The arena was indirectly illuminated with weak white light (30 lx in the center). Each mouse was put in a corner of the open field and allowed to explore the entire apparatus for 10 min. Video tracking software Ethovision (Noldus, Netherland) recorded and automatically analyzed the total distance traveled within the arena and the time spent in two distinct zones: the center and the periphery (sec). The apparatus was deep-cleaned between subjects using a 70% ethanol and water solution.

### 2.5. Cytokine level measurement: ELISA

Mouse peritoneal cell suspensions were collected 6 h after LPS intraperitoneal (IP) injection on C57Bl6/J pregnant mice. After mouse euthanasia with an anesthetic overdose (starting with an isoflurane flow rate of 7% and continuing isoflurane exposure until two minutes after breathing stopped), 3 mL of cold PBS was injected into the peritoneal cavity using a 27 G needle. The abdomen was gently massaged and then as much fluid as possible was collected using a 25 G needle attached to a 5 mL syringe. The collected sample was centrifuged at 2000 rpm for 10 min and the supernatant was collected for analysis. The levels of pro-inflammatory cytokine IL-1β and IL-6 (DuoSet® ELISA Development System, R&D Systems, Minneapolis, MN, USA) in the peritoneal wash sample were measured using ELISA kits according to the manufacturer’s protocol.

### 2.6. Statistical and data analysis

Data analysis was carried out using Graphpad Prism 9 (Graphpad Software, San Diego, CA, USA). Data are presented as mean ± SEM for each experimental group. Each point corresponds to an individual. As we are assessing effects on small samples (*n* < 30) and as our data do not follow a normal distribution, we analyzed them using non-parametric procedures specially adapted for these kinds of data. There are two main advantages of this statistical procedure compared to a parametric approach. First, these methods are not reliant on population assumptions, eliminating the risk of not satisfying prerequisite criteria. Second, they demonstrate improved power efficiency compared to larger sample sizes. Independent two-group comparisons were analyzed using the Mann–Whitney test. When more than two groups needed to be considered, the analysis was done using the Kruskal–Wallis “ANOVA-on-ranks” test, and then, when necessary, suitable post-hoc analysis (Dun test) was performed for intra-group comparison. When an analysis required repeated measures, a two-way ANOVA with repeated measures was performed. In order to analyze data compared to a hypothetical defined value, a one-sample Wilcoxon test was carried out. *p* values of ≤0.05 were considered statistically significant.

## 3. Results

### 3.1. A low maternal dose of LPS does not alter physiological parameters and maternal behavior

**Figure 3:**
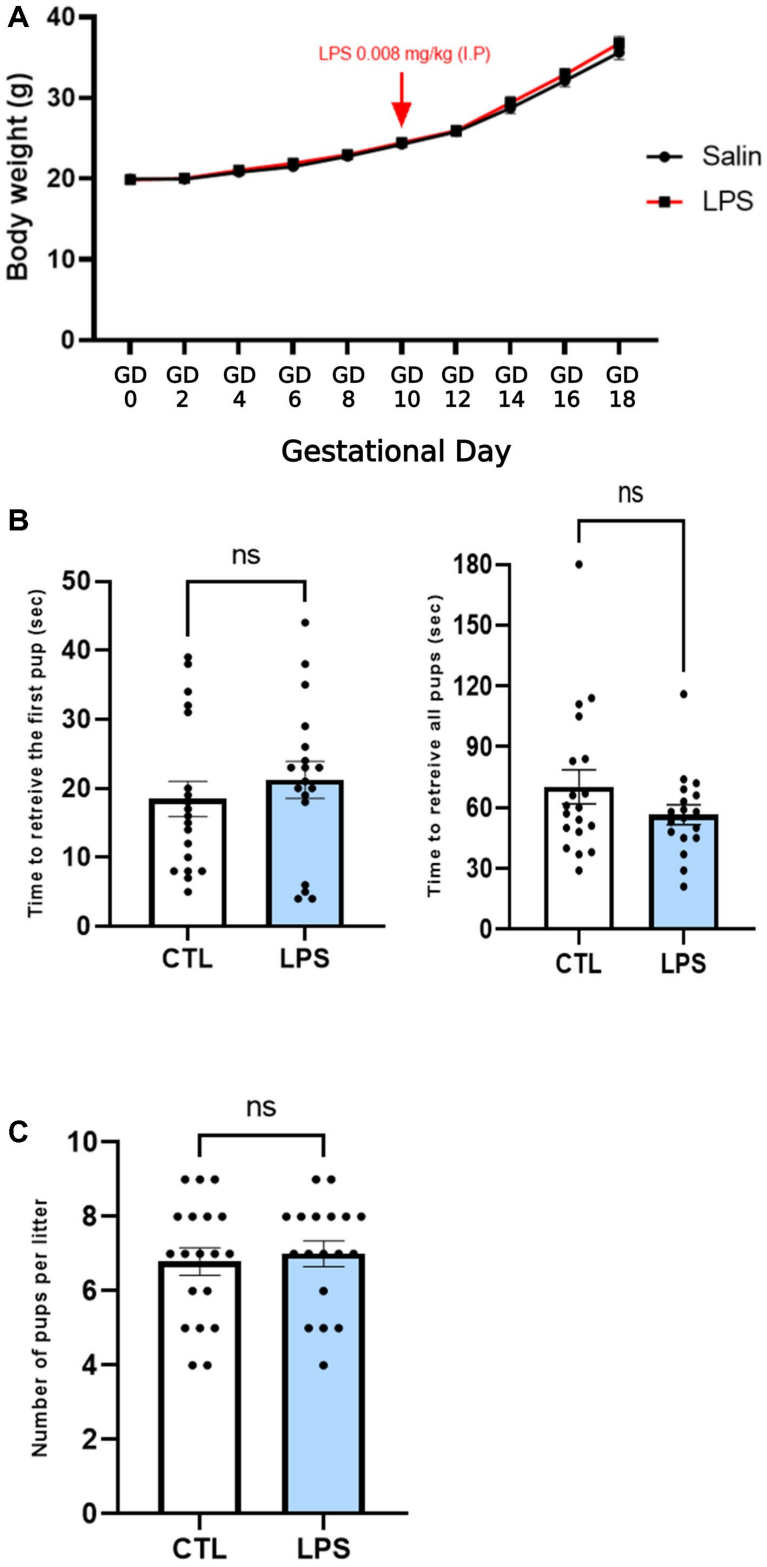
A low maternal dose of LPS does not alter physiological parameters or maternal behavior. Effect of an LPS maternal immune challenge at GD 10.5 (0.008 mg/kg) C57Bl/6 mice on body weight gain during pregnancy **(A)**, on maternal behavior in the pup retrieving task **(B)**, and on the number of pups per litter **(C)**. A low dose of LPS did not induce any changes to maternal body weight gain during pregnancy **(A)**. The latency to return one, followed by all isolated pups was recorded. No significant differences were observed between groups. Data are expressed as mean ± SEM. *N* = 19 (CTL), *N* = 18 (LPS). Comparisons were made by a two-way ANOVA **(A)** and Man-Whitney **(B,C)**.

An immune challenge of a 0.008 mg/kg dose of LPS did not induce any modification in the dams as reflected by the lack of effect on body weight gain during the gestational period (Figure 3A). Additionally, maternal behavior was not shown to be altered by an LPS immune challenge, as the time to retrieve the first pup and the mean time to retrieve all pups did not differ between the CTL and LPS-sensitized females (Figure 3B). Litter size was not affected either as there was no statistical difference in the mean number of pups per litter between the CTL and LPS groups (Figure 3C). In accordance with the literature, the low dose used did not disturb either maternal behavior or body weight.

### 3.2. A low dose of LPS in pregnant females induces a significant increase in pro-inflammatory cytokine levels

**Figure 4:**
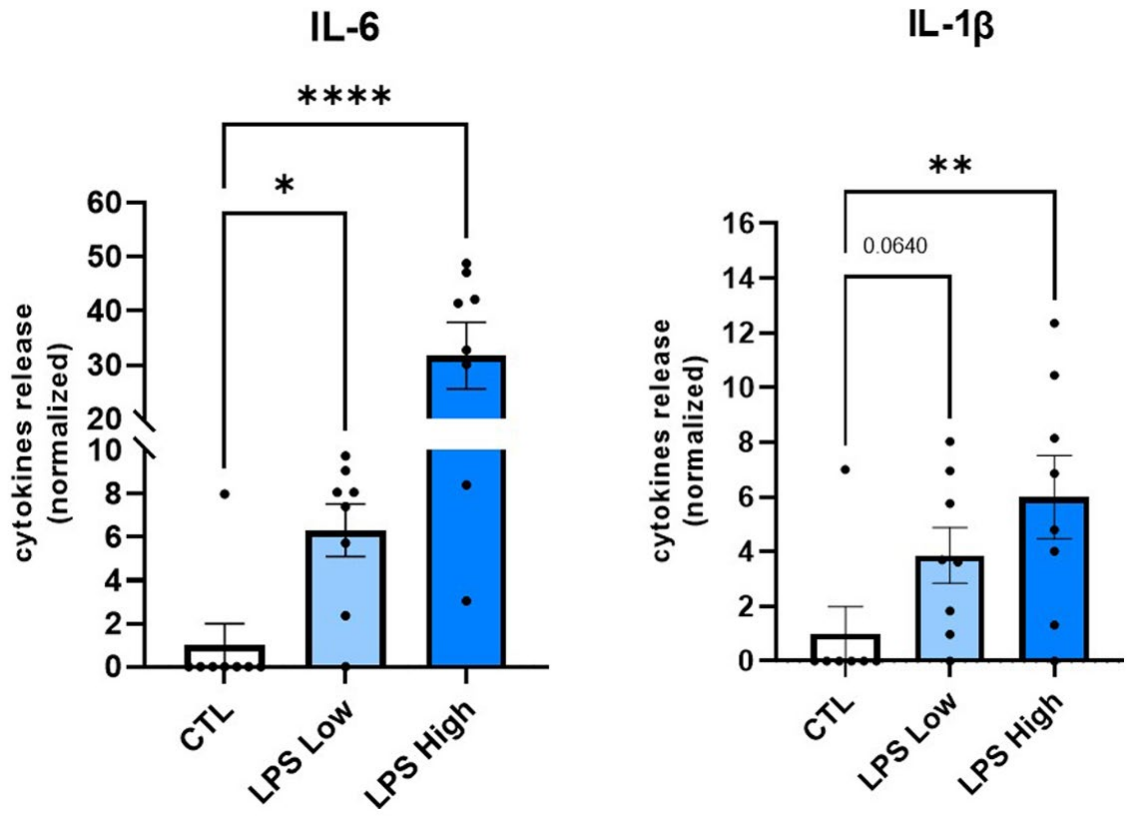
Low dose of LPS in pregnant females induces a significant increase in pro-inflammatory cytokine levels. Effect of the LPS prenatal immune challenge at GD 10.5 on pregnant C57Bl/6 mice on pro-inflammatory cytokine levels in the peritoneal wash. Levels of cytokines IL1-β and IL-6 released were measured by ELISA 6 h after an IP injection of LPS at 0,008 mg/kg (Low Dose). For the positive control, pregnant females received an IP injection of LPS at 0.1 mg/kg (High Dose). Data are expressed as mean ± SEM. *N* = 7 (CTL), *N* = 8 (LPS Low Dose), *N* = 8 (LPS High Dose). *p < 0.05, **p < 0.01, ***p < 0.001, ****p < 0.0001 (Kruskal Wallis) compared with Sal/CTL group.

We first aimed to determine whether a low-dose LPS prenatal immune challenge triggers the release of pro-inflammatory cytokines, such as IL-6 and IL-1ᵦ, which are major mediators of inflammation. ELISA analysis showed that an immune challenge at a low dose of 0.008 mg/kg induced an increase in IL-6 and IL-1ᵦ, in the peritoneal wash, 6 h after the injection (Figure 4). For IL-6, the level was approximately six times higher than the control, and a trend of approximately three times higher for IL-1ᵦ. As a positive control, females that received a higher dose of LPS (0.1 mg/kg), previously described as inducing a pro-inflammatory response, presented a significant release of both IL-6 and IL-1ᵦ, respectively, indicated by an increase of 5 and 30 times more than control (Figure 4). Thus, an LPS prenatal immune challenge at the low dose of 0.008 mg/kg induced a significant release of pro-inflammatory cytokines, even though physiological parameters and behavior were not disturbed.

### 3.3. For motor coordination, an asymptomatic maternal immune challenge sensitizes post-natal response to a low dose of pollutants

**Figure 5:**
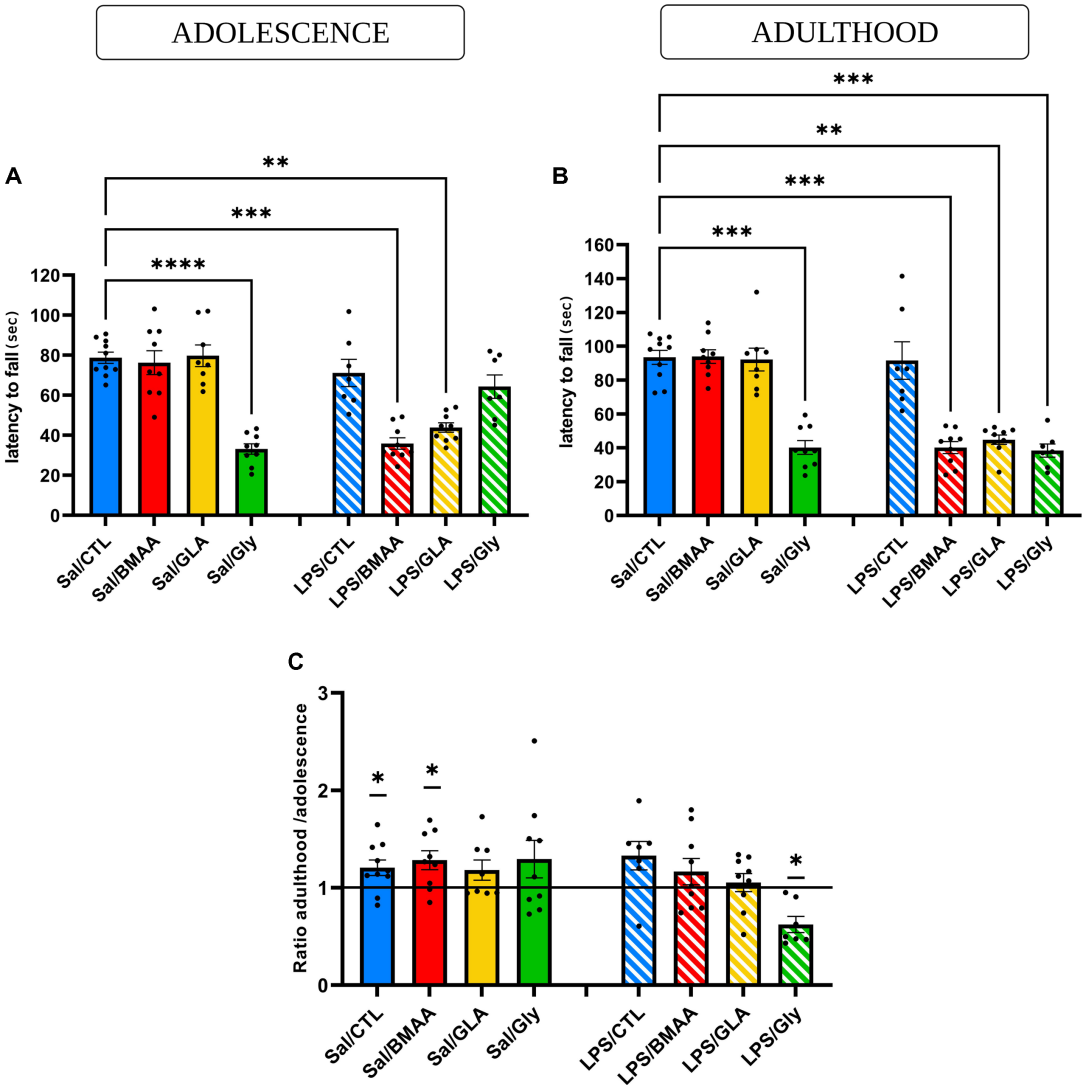
Asymptomatic maternal immune challenge sensitizes post-natal response to a low dose of pollutants – motor coordination. Effect of a prenatal immune challenge followed by postnatal chronic exposure to BMAA (50 mg/kg), GLA (0.2 mg/kg), or GLY (5 mg/kg) on the rotarod test in adolescence **(A)** and adulthood **(B)**. The balance and motor coordination of young and adult male mice were assessed in an accelerating mode of the rotarod test by measuring the latency to fall. Motor learning was represented in a ratio of latency to fall in adulthood and adolescence **(C)**. Data are expressed as mean ± SEM. *N* = 10 (Sal/CTL), *N* = 9 (Sal/BMAA), *N* = 8 (Sal/GLA), *N* = 9 (Sal/GLY), *N* = 7 (LPS/CTL), *N* = 9 (LPS/BMAA), *N* = 9, (LPS/GLA), *N* = 7 (LPS/GLY). **p < 0,01, ***p < 0,001, ****p < 0,0001 (Kruskal Wallis) compared with Sal/CTL group. *p < 0.05 (One sample Wilcoxon) compared to a hypothetical value of 1.

Balance and motor coordination were assessed in the accelerating mode of the rotarod test by measuring the latency to fall. During the adolescent period, neither offspring postnatally exposed to either BMAA or GLA, nor those that were prenatally exposed to a low dose of LPS, displayed an impairment to motor coordination (latency to fall was not different to the Sal/CTL group) (Figure 5A). Data showed that a single asymptomatic prenatal immune challenge was not enough to induce any modification in the latency to fall. However, individuals double-hit with a prenatal immune challenge, followed by chronic exposure to BMAA or GLA fell from the rod significantly faster than the Sal/CTL group (Figure 5A).

Later, during adulthood, the same profile was observed for this parameter, suggesting a long-term effect (Figure 5B). During adolescence, offspring subjected to postnatal exposure to GLY displayed a decrease in the latency to fall from the rotarod, but not after a prenatal immune challenge. In fact, after an asymptomatic prenatal LPS challenge, resilience was observed in the LPS/GLY group, with no significant differences compared to the Sal/CTL group (Figure 5A). However, later during adulthood, even though a postnatal exposure to GLY still impaired balance and motor coordination, prenatal LPS exposure sensitized a second postnatal offspring exposure to GLY, as the latency to fall became significantly different compared to the Sal/ CTL group (Figure 5B). In order to evaluate motor learning abilities between adolescence and adulthood, the ratio of latency to fall during adulthood and adolescence was calculated. Only the Sal/CTL and Sal/BMAA groups expressed motor learning, as the ratio was significantly different from the hypothetical value of 1. Mice from the LPS/GLY group displayed a ratio significantly lower than 1, illustrating a drop in motor coordination performance. Nevertheless, all other groups demonstrated insignificant differences between their performances in adolescence and later in adulthood, with a ratio statistically no different from 1 (Figure 5C). Thus, an asymptomatic prenatal immune challenge influenced the post-natal response to a low dose of environmental toxicants in terms of motor coordination performance and motor learning, with disruption occurring in adolescence and persisting into adulthood.

### 3.4. Asymptomatic maternal sensitization influences the post-natal response to a low dose of pollutants in terms of emotional and locomotor behavior

**Figure 6:**
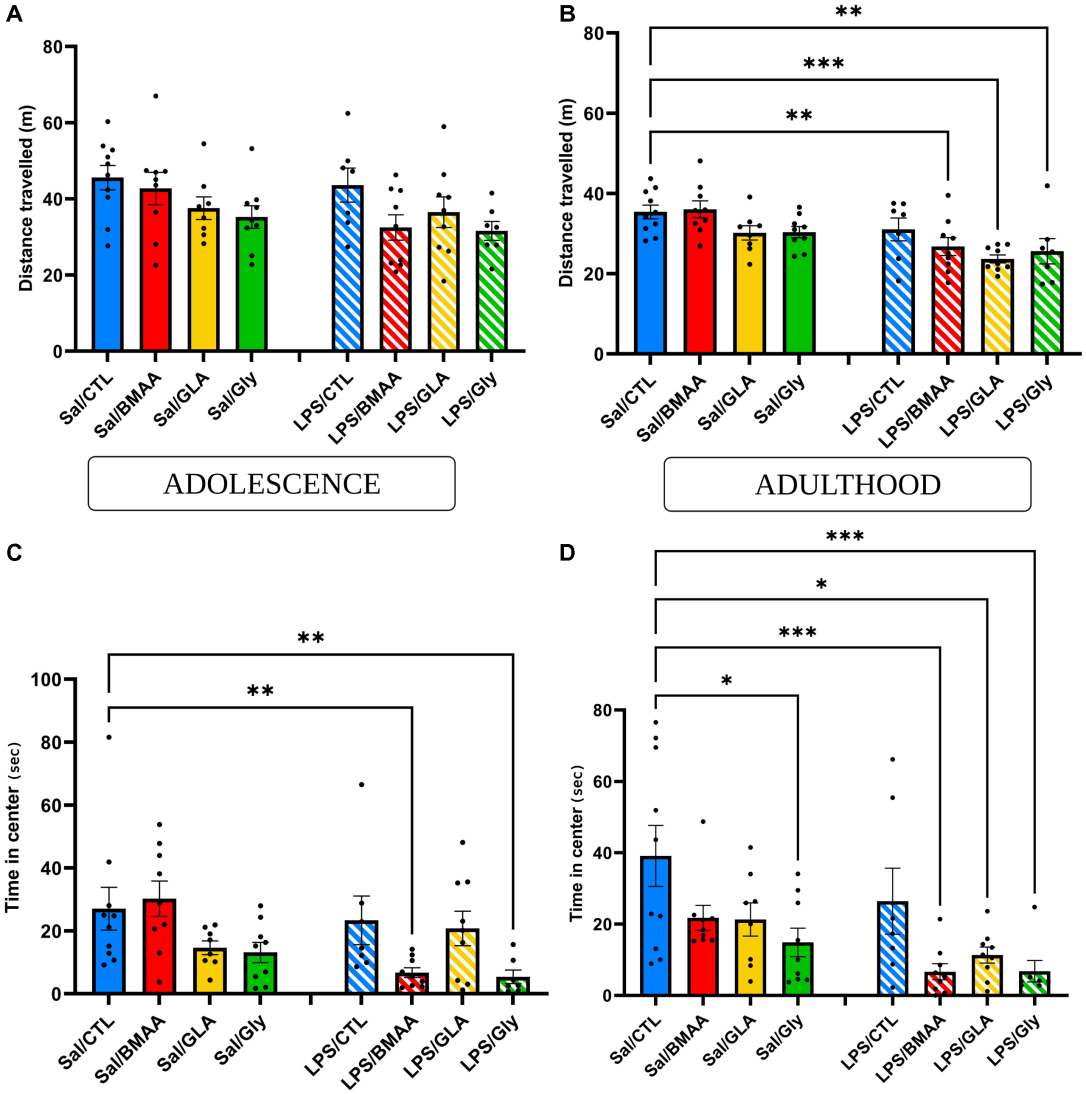
Asymptomatic maternal sensitization influences post-natal response to a low dose of pollutants - emotional and locomotor behavior. Evaluation of emotional parameters (time spent in the center) and locomotor activity during adolescence **(A,C)** and adulthood **(B,D)** in the open field. Maternal asymptomatic immune challenge influenced post-natal response to a low dose of environmental toxicants on locomotor activity **(A,B)** and emotional behavior **(C,D)**. Data are expressed as mean ± SEM. *N* = 10 (Sal/CTL), *N* = 9 (Sal/BMAA), *N* = 8 (Sal/GLA), *N* = 9 (Sal/GLY), *N* = 7 (LPS/CTL), *N* = 9 (LPS/BMAA), *N* = 9, (LPS/GLA), *N* = 7 (LPS/GLY). *p < 0,05, **p < 0,01, ***p < 0,001 (Kruskal Wallis) compared with Sal/CTL group.

Anxiety state and locomotor activity were tested using an open-field test. During adolescence, both mice postnatally exposed to either BMAA or GLY, and those that had been prenatally challenged to a low dose of LPS spent a similar amount of time in the center of the arena and traveled the same distance compared to the Sal/CTL group (Figures 6A,C). However, even though the total distance traveled was not impacted, individuals double-hit with a prenatal immune challenge followed by chronic exposure to BMAA or GLY spent significantly less time in the central zone of the open field. Moreover, post-natal exposure to GLA, whether combined with an asymptomatic prenatal immune challenge or not, did not induce any significant modification to these parameters (Figures 6A,C). During adulthood, neither offspring exposed to either BMAA, GLA, or GLY, nor those subjected to an LPS prenatal maternal immune challenge displayed any modification in general locomotor activity. The total duration spent in the central zone of the open field was not impacted in these groups except for those exposed postnatally to GLY (Sal/GLY vs. Sal/CTL *p* < 0.05*). Interestingly, the groups exposed to a low-dose LPS prenatal immune challenge, followed by postnatal exposure to BMAA, GLA or GLY, presented a decrease in both the amount of time spent in the center and total distance traveled (Figures 6B,D). Thus, a maternal asymptomatic immune challenge influenced the post-natal response to a low dose of environmental toxicants in terms of emotional behavior and locomotor activity.

## 4. Discussion

Converging evidence across epidemiological and experimental studies have identified prenatal maternal immune activation (MIA) and pre- or post-natal exposure to various xenobiotics as a risk factor for neurological disorders, including neurodegenerative diseases (Richardson et al., 2014; Bao et al., 2022). However, the biological processes are complex and the penetrance seems to be incomplete. It suggests that multiple exposures to several insults during the perinatal period should be necessary for the onset of these diseases (Kristin et al., 2019). The present work aimed to explore the concept of the “multiple-hit hypothesis.” Firstly, an MIA was induced by an asymptomatic dose of LPS. Next, a second hit consisted of chronic post-natal exposure to low doses of BMAA, GLA, or GLY. Our results suggest that either a prenatal LPS immune challenge or post-natal exposure to the xenobiotics BMAA or GLA alone are not sufficient to disturb behavioral performances in adolescence and later in adulthood. However, the double-hit exposure induced significant behavioral changes in offspring in adolescent and adult stages. Our work demonstrates that an asymptomatic maternal sensitization associated with a low-dose exposure to xenobiotics induces neurological disorders. This suggests a molecule dependent synergistic effect in line with the “multiple-hit hypothesis.”

Over the years, it has been well documented that maternal immune activation, mimed by LPS administration, among others, is a serious risk factor for developing neurological disorders such as neurodevelopmental disorders (Han et al., 2021). Indeed, in previous studies assessing LPS activation during gestation, doses usually needed to range from 0.02 to 0.1 mg/kg in order to induce neurodevelopmental disorders (Hsueh et al., 2017, 2018; Mouihate et al., 2019). However, at these higher doses, there are significant side effects on litter size, due to abortion, fetal death, or behavioral sickness in dams (Kirsten et al., 2010a,b). In the present work, we used a 0.008 mg/kg LPS dose, which is on average 10 to 13 times lower than those usually used in the literature (Boksa, 2010; Arsenault et al., 2014). Our goal was to induce low inflammation with an asymptomatic dose rather than major disturbances in the pregnant female (Le Belle et al., 2014). In our experimental conditions, we have shown that maternal behavior and physiological parameters such as body weight were not affected by the low LPS immune challenge, even though it induced a significant increase in the systemic pro-inflammatory cytokines IL-1β and IL-6 in dams. Based on data, during adolescence and later in adulthood, mice prenatally exposed to a low dose of LPS had no statistical deviations in general locomotor activity, anxiety-like, or motor coordination behavior. Interestingly, previous studies discussed the implication that an increase in IL-6 levels during pregnancy could have an impact on the cognitive behavior of offspring later in life (Samuelsson et al., 2006). Harsh maternal conditions, including MIA released mediators, may reach the developing fetus by crossing the placenta and then interfere with the ongoing developmental mechanisms of the fetal brain and cause brain disorders in the later life of the affected fetus (Meyer, 2014). In fact, it is clearly established that a maternal immune activating event due to LPS during pregnancy induces the increase of pro-inflammatory cytokines such as IL-1β, leading to short and long term effects in offspring (Rousset et al., 2006). Even though our data suggest that the circulating cytokines must reach a significant level in order to induce detectable neurodevelopmental defects, silent effects must not be excluded. In human studies, most maternal infections do not lead to neurological disorders in offspring (Estes and McAllister, 2016), suggesting a resilience effect or a protective/adapted response of the organism. In our experimental procedure, the prenatal LPS administration did not show behavioral disruption in offspring, validating our asymptomatic first hit approach.

In regards to our choice of xenobiotics, it has previously been established that exposure to BMAA, GLA, or GLY has major neurodevelopmental effects. Many epidemiological and preclinical studies have investigated the neurological outcomes of these compounds (Newell et al., 2022; Chang et al., 2023). Indeed, it has been shown that a BMAA post-natal exposure of 600 mg/kg leads to motor and cognitive impairments in adulthood (Karlsson et al., 2009b). Similarly, de Oliveira and her team showed that maternal exposure to 50 mg/kg of Glyphosate Based Herbicide (GBH) induced behavioral impairments and oxidative stress in offspring with neurodevelopmental disorders (de Oliveira et al., 2022). In regards to GLA exposure, previous data showed that maternal exposure to 2 mg/kg reduced locomotor activity and impaired memory in offspring (Dong et al., 2020). In agreement with previous studies, our strategy of using low doses of these molecules showed that mice chronically exposed to either BMAA or GLA during the post-natal period had no detrimental behavioral deviation in adolescence or later during adulthood (Table 1). However, few previous studies explore the effect of a low dose of these kinds of agents. Studies in rodents have shown that chronic exposure to a low dose of BMAA induces only a mild effect, if any, on behavioral performances both in adults and offspring (Cruz-Aguado et al., 2006; Laugeray et al., 2018). The disparity in these results has been discussed and can be explained in particular by the low transport of BMAA into adult rodent brains compared to the brains of neonatal rats (Karlsson et al., 2009a). Even though BMAA-related neurotoxicity has mainly been investigated from the perspective of neurodegeneration, few studies have reported that early exposure to a low dose of BMAA, especially during the neonatal period, may exert subtle neuroanatomical changes leading to behavioral alteration rather than neurodegeneration (Karlsson et al., 2015). In line with these findings, previous work conducted by our team demonstrated that perinatal exposure to GLA at 0.2 mg/kg led to neuroblastic migration disturbances but was not associated with behavioral impairment (Laugeray et al., 2014; Herzine et al., 2016). Compared to post-natal exposure to BMAA and GLA, mice chronically exposed to GLY during the same period displayed motor coordination deviation from adolescence that persisted later in adulthood. Other studies have already demonstrated that higher doses induce disturbances to locomotor activity and anxiety behaviors in rodents, for both adults and offspring (Hernández-Plata et al., 2015; Gallegos et al., 2016; Baier et al., 2017). In agreement with previous literature, it seems that high-level exposure to GLY may exert a deleterious neurological effect independent of the exposure period. However, to our knowledge, our current study is the first to evaluate offspring behavior after post-natal exposure to a low dose of GLY. Indeed, the GLY dose we assessed here is one-tenth of the current “No Observable Adverse Effect Level” (NOAEL), making the post-natal period particularly sensitive to GLY. These results, therefore, demonstrated that although these xenobiotics did not induce significant or major effects at these doses, a low dose GLY exposure leads unexpectedly to motor and anxiety behavior impairments.

**Table 1:** Summary table of post-natal behavioral effect on offspring during adolescent and adult periods after a multiple-hit protocol.

Adolescence	−LPS			+ LPS			
	BMAA	GLA	GLY	CTL	BMAA	GLA	GLY
Motor coordination	Ø	Ø	+ +	Ø	+ +	+ +	Ø
Locomotor activity	Ø	Ø	Ø	Ø	Ø	Ø	Ø
Anxiety state	Ø	Ø	Ø	Ø	+ +	Ø	+ +

Adulthood	−LPS			+ LPS			
	BMAA	GLA	GLY	CTL	BMAA	GLA	GLY
Motor coordination	Ø	Ø	+ +	Ø	+ +	+ +	+ +
Locomotor activity	Ø	Ø	Ø	Ø	+ +	+ +	+ +
Anxiety state	Ø	Ø	+ +	Ø	+ +	+ +	+ +

The ∅ symbol means that there is no significant effect. ++ means that there are significant modifications.

Our multiple-hit strategy shows that greater alterations to neurodevelopmental trajectories were observed in offspring exposed to both prenatal immune activating events and chronic post-natal xenobiotics. During adolescence, offspring prenatally subjected to a maternal immune sensitization and chronically exposed to BMAA displayed impairment in motor coordination and an increase in anxious behavior. These behavioral disturbances were still observed later during adulthood, as well as a decrease in general locomotor activity. In line with these results, the LPS/GLA group presented a deficit in motor coordination from adolescence, confirmed later in adulthood during which an increase in anxiety like-behavior was observed as well as a decrease in general locomotion. However, as described in Table 1, the effects induced by post-natal GLY exposure were not potentiated by maternal LPS sensitization (Table 1). Interestingly, we show that post-natal chronic exposure to low doses of either BMAA or GLA was not enough to induce behavioral disturbances in offspring. These findings demonstrate that a single risk factor may not be sufficient to cause enduring and long-lasting modifications in behavior. This is in accordance with a previous study conducted by Guma and her co-workers, which showed that mice either prenatally subjected to MIA in early gestation, or postnatally exposed to chronic THC during adolescence did not exhibit persistent behavioral abnormalities later in adulthood (Guma et al., 2022). This *in vivo* analysis revealed a cumulative effect of prenatal MIA and adolescent cannabis exposure on neurodevelopment, suggesting that “multiple hits,” within an individual are required for disease onset. Accordingly, in our experimental design using perinatal pollutant exposure, we also demonstrated a synergistic effect of two different hits, prenatal MIA succeeded by post-natal exposure to xenobiotics. Taking together, all results demonstrate that MIA sensitization depends on second hit nature, suggesting that several pathways could be affected and should be investigated in future research.

Recent clinical and preclinical findings suggest that MIA induces the dysregulation of fundamental neurodevelopmental programs. MIA, therefore, affects juvenile and adult brain responsiveness to cumulative exposure to harmful environmental insults during the juvenile period, adulthood, and aging (Krstic et al., 2012; Knuesel et al., 2014). These long-term impairments to brain function and behavior, therefore, often result from early life events. The early life of an individual is composed of multiple critical windows during which developing events occur. The pre-and post-natal periods are essential for brain development, and highly sensitive in terms of exposure to any environmental stimuli. The proliferation and differentiation of neuronal precursors, as well as the establishment of neuronal circuits, lead to the formation of the fundamental structure of the brain. This critical window of brain development, better known as the brain growth spurt (BGS) is also characterized by a significantly high rate of development and proliferation of glial cells, as well as the maturation and invasion of microglia in the spinal cord and the brain (Tay et al., 2017). In rodents, it occurs during the first 3–4 weeks after birth and the corresponding period in humans starts during the last trimester of pregnancy and continues until at least 2 years after birth (Dobbing and Sands, 1979). Thus, disturbances of these tightly regulated processes may negatively affect the establishment of basic neuronal circuits, weakening the fundamental structure of the brain. This could be the origin of permanent impairments, leading to a wide range of enduring adverse impacts later in life. It thus confers greater importance on the multiple-hit phenomenon, which needs to be investigated further, particularly during these highly sensitive periods of brain development. In this context, a more refined characterization of the permanent impairments would be necessary. The rotarod and open field tests allowed us to reveal the presence of motor-like disease symptoms. However, this initial observation would require further investigations using devices dedicated to motor phenotypes such as muscular strength, coordination defects, or ataxia (Brooks and Dunnett, 2009; Osmon et al., 2018). These additional investigations would lead to a better understanding of the long-term effects that may be associated with this type of disorder.

We found that exposure to a single risk factor, either prenatal immune sensitization to a low dose of LPS or chronic postnatal exposure to xenobiotics, is not sufficient to induce early and long-lasting behavioral changes in offspring. In contrast, exposure to both risk factors may induce early and persistent behavioral impairment. Indeed molecular, structural, or epigenetic modifications could be at the origin of an excessive reactivity to a further second hit. This posits MIA as a “disease primer” (Meyer, 2014) to make an individual more sensitive to subsequent environmental exposures in triggering disease-related symptoms later in life (Ayhan et al., 2016). While work focusing on interactions between MIA and environmental risk factors steadily increase, animal model outcomes suggest that even subclinical maternal infection can make offspring much more vulnerable to second “hits” (Knuesel et al., 2014). Sub-threshold MIA increases the likelihood of environmental risk factors, such as stress and drug use, causing neurodevelopmental phenotypes in offspring. It is important to underline that multiple stress exposure is common during the developmental period and most early life stressors are interdependent when it comes to inducing persistent neuroanatomical and behavioral disturbances. In fact, the timing of exposure is of paramount importance, as specific phenomena occur at different time points in brain development and induce differential effects. Thus, it is of paramount importance to better understand the cellular and molecular mechanisms underlying these impairments. In the last decade, an increasing number of studies have put forward the relevance of the involvement of the gut-brain axis. In fact, microbiome disruption can be induced by several environmental factors and could lead to neurological disorders (Stiemsma and Michels, 2018). Mesnage et al. showed that GLY and GBH inhibit the Shikimate pathway in plants. Even if this pathway is not found in animal cells, it is essential to the metabolism of some bacterial species found in human gut microbiota (Mesnage et al., 2021). Previous studies reported that chronic and sub-chronic exposure to GLY or GBH induces an alteration in gut microbiome composition, e.g., a decrease in levels of some specific microbes including *Lactobacillus, Bacteroïde* or *Firmicutes* (Bali et al., 2019). This GBH-dependent microbiome imbalance induces an increased production of pro-inflammatory cytokines and reactive oxygen species (ROS), which are associated with neurodevelopmental impairments (Barnett et al., 2022). In line with these findings, other studies have shown a developmental and functional alteration of the central nervous system (CNS) after changes in the intestinal environment induced by BMAA or GLA exposure (Dong et al., 2020; Esteves et al., 2023). Based on the impact of our three molecules on the microbiota, the data suggest disruptions of the gut-brain axis in our model of exposure. Further studies should be performed to help assess the potential connection between the microbiota and the consequences of “multiple-hit” exposure.

To conclude, our results show that asymptomatic maternal inflammation has a priming effect on subsequent exposure to low doses of xenobiotics in both motor coordination and locomotion. To our knowledge, this work is one of the first to use a longitudinal design extending from early pregnancy to adulthood to highlight the crucial importance of asymptomatic maternal inflammation in priming environmental contaminant-induced motor neuron disease-like symptoms. These findings raise an important public health issue. A comprehensive evaluation of the toxicological effects of pesticides and biotoxins is required and further clinical, pre-clinical, and epidemiological studies taking multiple exposures to several insults into account need to be performed. This work also paves the way for extra studies aiming at characterizing the biological pathways involved in these processes.

## Data availability statement

The raw data supporting the conclusions of this article will be made available by the authors, without undue reservation.

## Ethics statement

The animal study was reviewed and approved by Ethics Committee for Animal Experimentation of CNRS Campus Orleans (CCO), registered (N°3) by the French National Committee of Ethical Reflexion for Animal Experimentation, under N° CLE CCO 2015-1084.

## Author contributions

AO and SMo conceived the experiments. AO performed and analyzed the experimental data and prepared the manuscript. AO, AM, SMé, AL, and SMo discussed the results. SMo, GG, and AO provided funding. All authors read, approved the final manuscript, and agree to be accountable for the content of the present work.

## Funding

This work was supported by the National research Agency (ANR NeuroTEM ANR-18-CE34-0006) and by “Association pour la Recherche sur la Sclérose Latéral Amyotrophique et autres Maladies du Motononeurone (ARSLA)” (“Jeune chercheur ARSLA 2021” Subvention). This work was also supported by European funding in Région Centre-Val de Loire (FEDER No. EX016008 TARGET-EX). Those funding had no role in study design, data collection, analysis and interpretation of data, writing of the manuscript, the decision to submit the article for publication.

## Conflict of interest

The authors declare that the research was conducted in the absence of any commercial or financial relationships that could be construed as a potential conflict of interest.

## Publisher’s note

All claims expressed in this article are solely those of the authors and do not necessarily represent those of their affiliated organizations, or those of the publisher, the editors and the reviewers. Any product that may be evaluated in this article, or claim that may be made by its manufacturer, is not guaranteed or endorsed by the publisher.

## Abbreviations

MIA: Maternal immune activation
GLA: Glufosinate ammonium
BMAA: β-N-Methylamino-L-Alanine
GLY: Glyphosate
GBH: Glyphosate-based herbicide
DOHaD: Developmental Origins of Health and Disease
ALS: Amyotrophic lateral sclerosis
AD: Alzheimer’s disease
PDC: Parkinsonism-dementia complex
CNS: Central nervous system
PND: Post-natal day
GD: Gestational day
IP: Intraperitoneal
